# Teeth in Relation to Degeneracy

**Published:** 1903-01-15

**Authors:** H. C. Rutter

**Affiliations:** Columbus, Ohio


### TEETH IN RELATION TO DEGENERACY.

BY H. C. RUTTER, M.D., COLUMBUS, OHIO.

Read before the Ohio State Dental Society, December 3, 1902.

Bacon says in his essay "Deformity:" "Deformed persons are commonly even with nature, for as nature hath done ill by them, so do they by nature." We see therefore that certain characteristic traits were noticed in deformed persons, and mental obliquities associated with their physical defects long prior to the scientific study and classification of these abnormal physical changes.

Shakespeare has most vividly portrayed the co-relation of abnormal tooth development with other physical and mental indications of the degenerate. His illustration of this form of mental and physical degeneration has not been excelled, if equaled, to this day.

Richard of York, a miserable and deformed wretch, the most wicked and bloody of all English rulers, thus describes himself:

"For I have often heard my mother say,
I came into the world with my legs forward.
Had I not reason think ye, to make haste
And seek their ruin which usurped our right?
The midwife wondered and the women cried.
Oh, Jesus bless us, he is born with teeth.
And so I was, which plainly signified
That I should snarl and bite and play the dog.
Then since the Heavens have made my body so,
Let hell make crook'd my mind to answer."

And to give emphasis, if possible, to this picture of a degenerate body and mind, Henry is made to say to him:

"The mother felt more than a mother's pain,
And yet brought forth less than a mother's hope.
To-wit, an indigested and deformed lump.
Teeth hadst thou in thy head when thou wast born,
To signify thou cam'st to fight the world."

But it remained for comparatively recent writers to elaborate and classify and in part discover the reason for the obliquities so often noticed in the ancestors of deformed persons. We are indebted largely to the labors of Morel, the grand Dusulle, Craft-Ebing, Lombrosi and other more recent writers for our inspiration in the study of these most interesting phenomena.

The word "degeneration" may be defined for our purposes as being worse than one's ancestors. The term is only comparative. There may be many degrees of degeneration, as one may be only slightly worse than his ancestors, or very much worse. And so in the changes we look for we may find but slight deviation barely perceptible up in varying degrees to almost complete change in structure, shape, formation, position and function. Degeneration, as it is now accepted, means a deterioration of the very root of the human tree, and is likely to be exhibited to a greater or less degree in all of its branches.

It is likely to be indicated by a change for the worse in one's members or appendages of the body, and also in one or more faculties of the mind. Thus an arm or a leg may be dwarfed or deformed, and an eye or an ear, the nose or mouth disfigured in some way by reason of which it may not present a symmetrical appearance or be as correct functionally as that of its author. The mentality may be weakened, some one or more of the passions or impulses be either much stronger or the inhibitory force by which they are controlled much weaker than in the ancestry.

It must not be understood that all malformations are included in the accepted definition of degeneracy. Many pre-natal changes result in a changed development in some one particular part of the body without impairing in the slightest degree the normal and healthy balance of the system or the harmonious relationship of the different organs with each other. For example, the union of two fingers or toes, the presence of a hair-lip, a cleft palate, the over-riding of a tooth, etc., are not, standing alone, to be considered evidences of degeneration as the term is generally accepted.

These are often mere accidents, and although they render the individual worse than his ancestor in the one particular respect, are no proof whatever of the general deterioration which the term degeneration is used to designate. Some one of these accidents of development is not unfrequently found in men of the strongest physical and mental constitution. It is only when they are associated with other evidences of constitutional deterioration that they can be properly designated as stigmata of degeneration.

Of all the stigmata of degeneration none are more characteristic of certain conditions, perhaps, than the teeth. While all parts of the body may, under certain conditions, present abnormal condition and development, we have nothing more uniform and persistent than particular forms of departure from health in the shape and arrangement and disease of the teeth in certain degenerated results of heredity.

In this paper I shall only generalize as I do not wish to take up much of your time, and shall therefore say what I have to say rather dogmatically and cite the numerous authorities I have consulted on the subject.

These indications of degeneracy are usually observed in the teeth at a very early period. To use a paradox, the first appearance of the teeth in relation to degeneracy is their non-appearance, or much-delayed appearance, as in rickets. While rickets has been recognized generally as a disease due to mal-nutrition and unsanitary surroundings, late researches prove that it is associated with degeneration and pre-natal changes and should be assigned a place along with afflications known as degenerative, such as idiocy, scrofula, etc. The eruption of the teeth in rickets is usually delayed and they are irregular in shape and size. The gums are often so soft and spongy and bleed so readily that the disease has been confounded with scurvy. In idiocy, cretinism and feeble-minded children, the teeth usually indicate the degenerative condition very early.

A visit to one of our idiotic asylums or to the hospital for epileptics will show an astonishing assortment of abnormal tooth development in patients where the disease is the result of degeneration and not merely accidental. They are irregular in shape. position and size, and decay very early in life. In the degenerate condition caused by inherited syphilis, the teeth furnish the most reliable and uniform guide to a knowledge of the true condition. This appearance was first observed and described by Johanthan Hutchinson, who says in relation to it:

"It consists in the dwarfing of the tooth, which is usually both narrow and short, and in the atrophy of its middle lobe. This atrophy leaves a single broad notch, vertical in the edge of the tooth, and sometimes from which notch a shallow furrow passes upward in both anterior and posterior surfaces, nearly to the gum. This notching is usually symmetrical. It may vary in degree in different cases, sometimes the teeth diverge and at other times slant toward each other."

Many abnormal conditions of the teeth are found in congenital epileptics, feeble-minded and idiotic. The teeth are mostly dwarfed. In some instances the space is wide and irregular. Often even the incisors are found decayed and broken off before puberty. The size and shape of the teeth, and their position, frequently protruding forward so as to elevate the upper lip and push out the lower lip, and give a horribly grotesque appearance to the degenerate. I well remember how the dreams of my younger boyhood were poisoned by a neighboring degenerate whose canine teeth protruded through his lips like the great tushes of a wild boar.

Of course this feature is frequently accentuated by unduly short lips, which in turn are often the stigmata of degeneration. It is important to remember that the relationship between diseased teeth and many other diseases is quite intimate, and that they mutually re-act the one against the other. That is to say that while in many cases ill health and heredity cause bad teeth, bad teeth often cause ill health and aggravate the ills resulting from degeneracy. Many nervous disorders are the direct result of defective teeth which in turn may have resulted from some inherited weakness of nutrition. Several well authenticated recoveries from epilepsy have resulted from proper treatment of defective teeth, and my experience goes to show that in nearly all cases that disease may be greatly benefited and results enormously ameliorated by the dentist. And so it is in many cases of neurasthenia, where a raging tooth or a lot of irritable teeth causes confusion of the entire nervous system and finally brings about such pathological changes as fasten upon the sufferer a permanent and incurable malady. Thus the defective teeth may stand as a direct cause of degeneration, the neurasthenia parent who in turn gives the world a degenerate offspring.

I shall not dwell upon the immediate results of carious teeth, that is, the effect upon the victim during periods of recrudescence, or the additional labor a frequent return of those periods impose upon the recording angel. Although I am inclined to the belief that a dentist never sees a real case of toothache. It is always my experience that some occult influence emanates from the dentist which relieves the pain before we actually come into his presence. I have carried the most torturing toothache to the very threshold of the dental office, when, presto, it disappeared, nor could I tell which tooth was sore or which side of the face had been painful. But it requires no great effort of the imagination to picture the effect upon the offspring of one suffering from the nervous strain imposed by chronic toothache. Perhaps no other ailment to which human flesh is heir is so exasperating, so destructive of moral and mental quietude, so harrowing in its effects upon the entire nervous system as the toothache, and all so little liable to receive from friends the sympathy which often soothes and softens other distressing maladies.

"When fever burns or ague freezes,
Rheumatics gnaw, or colic squeezes,
Our neighbor's sympathies may ease us,
We pitying moan,
But thee, thou hell of all diseases, ay mocks our groan."

To sum up, if I should be asked what is the most prolific cause, remote and immediate, of insanity, idiocy, epilepsy and degeneracy, I should unhesitatingly reply, primarily indigestion. And we all recognize the prominent part played by the teeth, indigestion and assimilation. It therefore follows that bond of union between the dentist and the neurologist must of necessity be close. Bad teeth do their primary effect in causing neurasthenia and nervous weakness and secondarily by impairment of digestion lead to degeneration in the offspring. These degenerate offspring propagate bad teeth in their children, the cycle therefore is complete, as it is in all natural phenomena. It requires heat to produce cold, which in turn gives us back heat. It requires water to render possible the growth of verdure on the arid plains, and in return that verdure brings bountiful rains. It requires force to produce the golden grain, which returns to us that force in a thousand-fold measure.

The lesson is easy; it is your province to give the world good teeth, and good teeth will in return give the world good men.

### DISCUSSION.

Dr. E. S. Talbot: I do not think there is anything in the doctor's paper which I could criticise. The doctor has had about twenty-five year's experience in connection with our public institutions, and knows whereof he speaks. I can only extend the work he has done, and give some examples of degenerate teeth, as seen in my practice. I have made my own definition of degeneracy because I think it is more applicable to the work which I have been doing for the past thirty years. It is this: A degenerate is one whose nervous system is unstable, and whose physical development is a departure from the normal. A degenerate organ is one which manifests either arrest of development or excessive development.

To show the presence of this condition, it is necessary for us to know something about evolution. Aristotle first noticed differences in development of tissue. He laid down the law of growth as that law by virtue of which an organ is lost for the good of the whole organism. Others taking their cue from this statement, changed the idea by stating that there is an arrest of development, or excessive development. A change in the human face is taking place. The brain is developing and the jaws and teeth are retrograding. Whenever disease affects an unstable nervous system, the jaws and teeth are affected because they are unstable. Of all the structures of the face, the teeth are most affected. An arrest of development is nearly always an atavism because the human body has developed through the lower forms of animal life to the higher, and an arrest of development is nearly always a return to some lower form of life.

Camper called attention to the development of the face of the chimpanzee on the one extreme ; to that of the typical Greek profile on the other. In the lower forms of life the jaws are larger in proportion to the brain. As we pass to the higher forms the brain pushes forward until at last, brain and jaws are on a perpendicular line. Here we commence our work. Here we commence to notice degeneration. We get deformity among the lower orders of life. It is only when the forehead has come forward that we find deformities of the face, jaws and teeth. In one form of arrested development the forehead is developed, but the development of the lower jaw is so far arrested that it talls entirely inside the perpendicular line dropped from the forehead. In such cases we have deformities of the nose.

In other individuals there is an arrest of development of the face, and the lower jaw has developed forward. This is not unusual. It is an atavism, a return to the original lower animal. It is important to note the difference between heredity and atavism. By virtue of heredity the child inherits directly from the parent. By virtue of atavism he inherits something remote.

On account of a defective liver, oxidation is not complete, the child grows up fleshy, and there is a large head, but the development of the face is arrested. You have all seen such cases. There is always an unstable nervous system accompanying such cases.

When we have arrest of development of the face, one of the jaws is affected; sometimes it is the upper, sometimes the lower. You have all seen cases of prominent upper teeth, the teeth have pushed forward in the process, but the jaw above the apices shows arrest of development.

Another common form of degeneracy is the V-shaped arch. This is a return to the reptilian type. It could not assume any other form, but I have not time to-night to show why this is. Another common form of degeneracy is the saddle-shaped arch. This is a return to the carnivorous type. Like the V-shape, it could not take any other form. Every form of degeneracy is a departure from one of these forms and is produced by the teeth when they come into the jaw.

By comparison with the normal vault, the degenerate vault will be seen to be high.

One of the causes of interstitial gingivitis is an arrest of development of the jaws and narrow processes.

When there is an unstable nervous system the process becomes hypertrophical. Prendergast, who murdered Carter Harrison, was a degenerate, there was arrest of the development of the upper jaw, with excessive prominence of the lower. The development of the brain was arrested. The right side of his head was much larger than the left.

In the lower vertebrates there are from forty to seventy-two teeth. Sometimes we find a tendency to return to this condition, with the result that there are extra teeth. It is singular that when this occurs the extra teeth are found in either the anterior part of the mouth or the posterior, and sometimes they are found in both at once. This is an atavism in two ways; it is an effort to return to the original number of teeth, and you will find by noting the shape of the extra teeth, that they endeavor to return to the cone shape of the lower order.

There are two theories as to the evolution of the teeth; one is the compressant theory, it holds that two of the original cone-shaped teeth have been pressed together to form a bicuspid and four to form a molar; the other is the theory which holds thatt here has been a budding from the original cone-shaped tooth. I think it can be shown that both theories are partly correct. If you will examine the syphilitic teeth due to development from disease, you will see that development has been arrested while they were trying to take on the cone shape.

In degenerates we always find pre-maxillary bones; we never find them in any other class of people. They mark a return to a lower form. Ridges through the roof of the mouth mark degeneracy. Sometimes they amount to lumps of bone. Sometimes union between halves of a jaw does not take place until the age of twenty-five or thirty. As a result of mastication, irritation is set up, resulting in the formation of a ridge.

